# Two Different Missense *C1S* Mutations, Associated to Periodontal Ehlers-Danlos Syndrome, Lead to Identical Molecular Outcomes

**DOI:** 10.3389/fimmu.2019.02962

**Published:** 2019-12-18

**Authors:** Isabelle Bally, Fabien Dalonneau, Anne Chouquet, Rebekka Gröbner, Albert Amberger, Ines Kapferer-Seebacher, Heribert Stoiber, Johannes Zschocke, Nicole M. Thielens, Véronique Rossi, Christine Gaboriaud

**Affiliations:** ^1^University Grenoble Alpes, CEA, CNRS, IBS, Grenoble, France; ^2^Division of Human Genetics, Institute of Human Genetics, Medical University Innsbruck, Innsbruck, Austria; ^3^Department for Operative and Restorative Dentistry, Medical University Innsbruck, Innsbruck, Austria; ^4^Institute of Virology, Medical University Innsbruck, Innsbruck, Austria

**Keywords:** C1s protease, complement system, periodontal, Ehlers-Danlos, HMGB1

## Abstract

Ehlers-Danlos syndromes (EDS) are clinically and genetically heterogeneous disorders characterized by soft connective tissue alteration like joint hypermobility and skin hyper-extensibility. We previously identified heterozygous missense mutations in the *C1R* and *C1S* genes, coding for the complement C1 proteases, in patients affected by periodontal EDS, a specific EDS subtype hallmarked by early severe periodontitis leading to premature loss of teeth and connective tissue alterations. Up to now, there is no clear molecular link relating the nominal role of the C1r and C1s proteases, which is to activate the classical complement pathway, to these heterogeneous symptoms of periodontal EDS syndrome. We aim therefore to elucidate the functional effect of these mutations, at the molecular and enzymatic levels. To explore the molecular consequences, a set of cell transfection experiments, recombinant protein purification, mass spectroscopy and N-terminal analyses have been performed. Focusing on the results obtained on two different *C1S* variants, namely p.Val316del and p.Cys294Arg, we show that HEK293-F cells stably transfected with the corresponding C1s variant plasmids, unexpectedly, do not secrete the full-length mutated C1s, but only a truncated Fg40 fragment of 40 kDa, produced at very low levels. Detailed analyses of the Fg40 fragments purified for the two C1s variants show that they are identical, which was also unexpected. This suggests that local misfolding of the CCP1 module containing the patient mutation exposes a novel cleavage site, between Lys353 and Cys354, which is not normally accessible. The mutation-induced Fg40 fragment contains the intact C-terminal serine protease domain but not the N-terminal domain mediating C1s interaction with the other C1 subunits, C1r, and C1q. Thus, Fg40 enzymatic activity escapes the normal physiological control of C1s activity within C1, potentially providing a loss-of-control. Comparative enzymatic analyses show that Fg40 retains the native esterolytic activity of C1s, as well as its cleavage efficiency toward the ancillary alarmin HMGB1 substrate, for example, whereas the nominal complement C4 activation cleavage is impaired. These new results open the way to further molecular explorations possibly involving subsidiary C1s targets.

## Introduction

Ehlers-Danlos syndromes (EDS) are a clinically and genetically heterogeneous group of heritable connective tissue disorders characterized by soft connective tissue alterations such as joint hypermobility, skin hyperextensibility, and tissue fragility (1). We previously identified heterozygous missense mutations in the *C1R* and *C1S* genes in patients affected by periodontal EDS (pEDS, OMIM 130080 and 617174) (2), a rare specific EDS subtype hallmarked by early severe periodontitis leading to premature loss of teeth (3), in addition to connective tissue alterations (4). The disease is inherited in an autosomal dominant manner and prevalence of pEDS is unknown. There is no evidence for recurrence of any pEDS variant. All pathogenic *C1S* variants were specific for individual families and are not listed in ExAc or gnomAD.

C1r and C1s are homologous serine proteases mainly known for their key role in activating complement through the classical pathway (CP) (5). Complement is a complex innate immune surveillance system orchestrating the elimination of pathogens, as well as immunological and inflammatory processes (6). In this context, C1r and C1s are homologous proteases associated to the C1q recognition unit, forming C1, the first component of the complement system identified. When antibodies (IgG or IgM) bind to surface antigens, a very potent activation signal for C1 becomes exposed in their Fc region. A cascade of proteolytic events is initiated, starting with the autoactivation of C1r, which then triggers the activation cleavage of C1s. Activated C1s in turn mediates the cleavage of C4 and then C2 to generate the CP C3 convertase, which can cleave the more abundant and central complement C3 component. These C1r and C1s activities are under tight physiological control by C1-inhibitor (5, 6). Moreover, the triggering of their activities within C1 is restrained by C1q specific surface targeting.

Up to now, a molecular link is missing, which would directly relate the nominal function of CP activation for the C1r and C1s proteases to the pEDS disease and its various heterogeneous-especially connective tissue-symptoms. Recent clinical observations and pre-clinical studies collectively suggest however that complement is often hyper-activated in periodontitis, one of the major symptom of pEDS, and that complement inhibition provides a therapeutic benefit for periodontal diseases (7). A reciprocally reinforced interplay between bacterial dysbiosis in periodontitis-associated biofilms, in favor of inflammophilic pathogens, and destructive inflammation enters a vicious circle in this context of excessive complement activation (7). Severe periodontitis in pEDS can proceed however without the classical periodontal pocketing (4), suggesting that the details of the pathological process might somehow differ.

Our aim is therefore to better understand the possible consequences of pEDS patient *C1S* variants at the molecular and enzymatic levels, with a special focus here on *C1S* variants initially identified, as listed in Table 1 (2). We will describe the main experimental steps that provide more support to the notion that these *C1S* mutations contribute to pEDS pathology. It starts from the observation that these mutations lead to the secretion of unexpected truncated C1s fragments. The detailed identification of the purified fragments then reveals that they turn to be identical for the two different C1s variants. Finally the functional characterization of their enzymatic activities shows how the mutations impact the canonical role of complement C4 activation, which opens further exploration and suggestions on possible subsidiary pEDS-related C1s targets.

**Table 1:** *C1S* variants analyzed in this study.

**DNA (c.)(GRCh38)**	**Protein (p.)**	**Domain**	**MAF**	**PROVEAN prediction**	**ClinVar**
c.880T>C	p.Cys294Arg	CCP1	Not listed	Deleterious (−10.095)	VCV000267349
c.945-947del	p.Val316del	CCP1	Not listed	Deleterious (−8.937)	VCV000267348

*PROVEAN (http://provean.jcvi.org) prediction tool sets a cutoff at −2.5 for “deleterious.” MAF, minor allele frequency*.

## Materials and Methods

### Proteins and Reagents

Oligonucleotides were purchased from Eurogentec, restriction and modification enzymes from New England Biolabs. C1s Fg40 and pEDS variants were engineered using site-directed mutagenesis with the QuickChange II XL kit (Agilent Technologies). N_α_-Benzoyl-L-arginine ethyl ester hydrochloride (Bz-Arg-OEt, BAEe) and ANTI-FLAG® M1 Agarose Affinity Gel were purchased from Sigma-Aldrich. HiTrap affinity columns were from GE Healthcare Life sciences, France. The following complement proteins were purified from human plasma according to published procedures (8, 9): C4, C1 inhibitor and activated C1r. For protein quantification, the following absorbance coefficient A_1%, 1cm_ at 280 nm and molecular weight (Mw) were used: C1r (12.4 and 86,300), C4 (8.3 and 205,000), C1inh (4.5 and 104,000) (8, 10), respectively.

### Production of C1s Variants in Mammalian Cells and Purification

**Figure 1:**
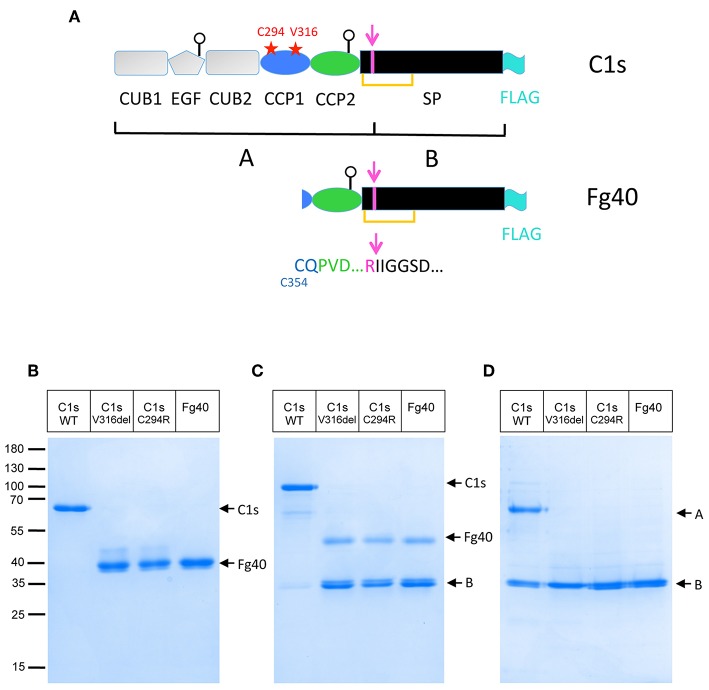
Truncated C1s fragments are secreted as a consequence of the two pEDS *C1S* mutations. **(A)** C1s modular structure showing the position of the two patient mutations (red stars) as well as the main C1s post-translational modifications discussed in the text: the activation cleavage site (magenta), leading to two chains (A,B) bridged by a SS bond (orange line), the two glycosylation sites on EGF and CCP2 (white circles) and the FLAG-tag added for purification (cyan). **(B,C)** SDS-PAGE analysis of 4 μg FLAG-affinity purified C1s WT and variants: C1sWT, mutants C1sC294R and C1sV316del, and Fg40 in non-reducing **(B)** and reducing conditions **(C)**. **(D)** The same samples after activation by C1r (as described in Material and Method section) in reducing conditions. Molecular masses (in kDa) of standard proteins are indicated on the left side of the gel **(B)**.

**Figure 2:**
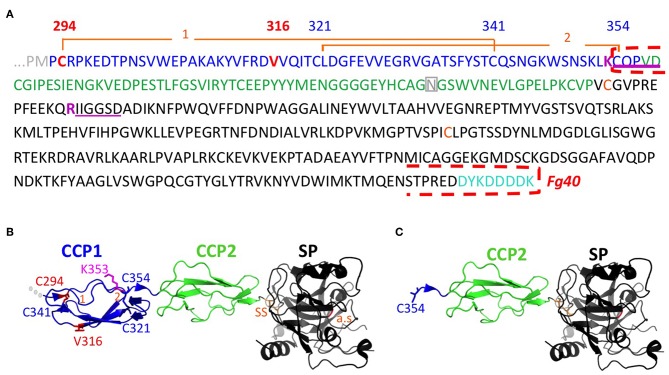
Sequence and structural details associated to the Fg40 fragment interpretation. **(A)** Sequence of C1s starting at the transition from CUB2 (gray) to CCP1 (blue), followed by CCP2 (green), and the serine protease SP domain (black). The mutations in CCP1 are shown in red. N-terminal sequences identified are underlined in magenta, indicating cleavage after the preceding basic residue (magenta). The N-terminal and C-terminal limits of Fg40 are indicated by red dashed brackets. Two disulfide bonds, C294-C341 (1) and C321-C354 (2), hold the structure of the CCP1 module. The two cysteines of the interchain disulfide bridge (SS, orange), the N406 glycosylation site in CCP2 (gray), and the C-terminal FLAG-tag (cyan) are also highlighted. **(B)** Native structure of the corresponding region in C1s (PDB code: 4j1y). **(C)** Fg40 fragment, as extracted from the native structure, showing its free N-terminal C354. Cleavage after K353 cannot occur in the context of the native structure shown in B. The active site (a.s., orange) is located in the SP domain, remaining intact in Fg40.

pcDNA3.1/Zeo C1sWT was obtained by insertion of a PCR amplicon of pFastBac1/C1s (11) in pcDNA 3.1/Zeo between *Nhe*I and *EcoR*I. For purification purposes a FLAG-Tag was also inserted on the 3'-end (12). Variants pcDNA3.1/Zeo C1sV316del, pcDNA3.1/Zeo C1sC294R, and pcDNA3.1/Zeo C1s Fg40 fragment were engineered by site directed mutagenesis. C1s Fg40 is the C-terminal fragment starting at Cys354, as illustrated in Figures 1A, 2A. The primers used for the PCR are listed in Figure S1.

The pcDNA3.1 C1s constructs were used for stable transfection of mammalian HEK293F cells and secreted proteins were then purified from cell culture supernatants on a 2.5 mL ANTI-FLAG M1 agarose column as described for the recombinant C1s-C1r-C1r-C1s tetramer (12). The concentration of the purified C1s proteins was estimated using the absorption coefficient A_1%, 1cm_ at 280 nm calculated using the PROTPARAM program on the Expasy Server (http://web.expasy.org/protparam/), and the experimental molecular mass. The parameters used were as follows, for WT C1s-FLAG (C1sWT): Mw 80,665, A_1%, 1cm_ at 280 nm 14.5, and for C1s variants C1sC294R / C1sV316del / Fg40: Mw 40,050, A_1%, 1cm_ at 280 nm 17.3.

### N-Terminal Sequencing and Mass Spectrometry (MS) Analyses

Amino acid sequence determination based on Edman degradation was performed using an Applied Biosystems gas-phase sequencer model 492 (s/n: 9510287J). Chromatography was used to identify and quantify the Phenylthiohydantoin-derivatized amino acid removed at each sequence cycle with an Applied Biosystems Model 140C HPLC system using the data analysis system for protein sequencing from Applied Biosystems (software Procise PC v2.1). Retention times and integration values of peaks were compared to the chromatographic profile obtained for a standard mixture of derivatized amino acids, using the PTH-amino acid standard kit (Applied Biosystems P/N 4340968).

Experimental molecular masses were deduced from MALDI TOF mass spectra, measured with an Autoflex mass spectrometer (Bruker Daltonics, Bremen, Germany) operated in linear positive ion mode. External mass calibration of the instrument was done using protein calibration standards from Bruker Daltonics providing mass accuracy < 1,000 ppm. Before mass analysis the protein samples (concentration: 7.5 μM in Tris 50 mM, NaCl 150 mM, pH 7.4) were mixed in a ratio 1:2, 1:5 or 1:10 (v:v) with sinapinic acid matrix (Sigma; 10 mg/mL in water/acetonitrile/trifluoroacetic acid, 50/50/0.1, v/v/v) and 1–2 μL of this mixture were deposited on the target and allowed to air dry. Mass spectra data were processed with Flexanalysis software (v.3.0, Bruker Daltonics).

### Production of HMGB1 in *E. coli* and Purification

*Escherichia coli* BL21(DE3) cells were transfected with the pET-28a plasmid coding for High mobility group protein B1 (HMGB1) with a C-terminal HisTag and optimized for bacterial production (13). HMGB1 was expressed using an autoinduction protocol (14). HMGB1 purification involves three steps. First, HMGB1 was affinity purified on a hiTRAP chelating HP column, eluted by a 10–500 mM imidazole gradient in 20 mM Tris, 150 mM NaCl, pH 8.0. A hiTRAP heparin affinity column was then used to remove the contaminating DNA bound to the HMG boxes, with a gradient from 0.15 to 1 M NaCl in 20 mM Tris, pH 8.0. A final step of gel filtration was performed using a superdex 75 column, in Tris 50 mM, NaCl 150 mM, pH 7.5. Protein concentration was estimated using a Mw of 26.6 kDa as determined by MS and a A_1%, 1cm_ at 280 nm of 8.11.

### Determination of the C1s Constructs Enzymatic Activities

Activation of proenzyme recombinant C1sWT and pEDS-related constructs was achieved by incubation of the purified proteins with active serum-derived C1r (C1r/protein molar ratio of 2.2%) for 100 min at 37°C, in 50 mM Tris, 150 mM NaCl, pH 7.4.

The esterolytic activity of activated C1s (WT and its various fragments) was measured against Bz-Arg-OEt, using an alcohol dehydrogenase/NAD-coupled spectrophotometric test, as described previously (8). BAEe (1 mM) was incubated with 2 to 10 picomoles of C1sWT or variants/fragments at 30°C and the absorbance at 340 nm was monitored during 10 min. The slopes were then plotted for each enzyme quantity and the overall activity in amount of BAEe transformed per second and per mole of enzyme was calculated.

The proteolytic activity on C4 was measured as reported previously (15). Briefly, C1sWT and its variants (0.002 μM) were incubated 10 min at 37°C with C4 (2.5 μM). A C1sWT concentration of 0.5 μM was used as a positive control. All kinetics were conducted in 150 mM NaCl, 5 mM EDTA, 20 mM sodium phosphate, pH 7.4. The reaction efficiency was measured by the quantification of the C4 fragments by SDS-PAGE analysis performed in reducing conditions.

The inhibition of the activated proteases by C1 inhibitor was achieved by incubation of the enzymes with a 2-fold molar excess of C1 inhibitor for 90 min at 37°C in 50 mM Tris, 150 mM NaCl, pH 7.4. The mixture was then used for proteolytic cleavage in the conditions specific to each enzymatic test.

HMGB1 (2 μg) was submitted to proteolysis by activated C1s (WT and pEDS-related variants) using a 4% molar enzyme/substrate (E/S) ratio in 50 mM Tris, 150 mM NaCl, pH 7.4, for 100 min at 37°C. The reaction products were then analyzed by SDS-PAGE in reducing conditions.

## Results and Discussion

### Truncated C1s Fragments Are Secreted at Low Levels for the Two pEDS Patient Variants

Two different *C1S* mutations have been initially identified in pEDS patients: the deletion of Val316 (p.Val316del) and the substitution of Cys294 into Arg (p.Cys294Arg), both located in the CCP1 module (Figure 1A) (2). Clinical symptoms of affected individuals were described elsewhere (2). In brief, individuals presented with mild elastic skin, easy bruising, fragile skin on fingers, and legs with pretibial discoloration and early severe periodontitis leading to tooth loss in the teens. The patients mutations were introduced in a recombinant pcDNA3.1 plasmid coding for C1s and transfected into HEK293-F cells in order to observe how they would affect the synthesis or secretion of the C1s protein variant. The initial transient transfection experiments lead to barely detectable secretion of the two C1s variants. However, careful observations lead to the detection of a faint main band at about 40 kDa, which has no counterpart in C1sWT. Since this fragment was secreted at very low levels, stable cell lines expressing these two C1s variants and C1sWT were generated in order to purify, analyse and compare the secreted products. A main band at about 40 kDa, which will be named thereafter Fg40, was reproducibly obtained (see Figure 1). The proteins were purified by one-step affinity chromatography, taking advantage of a C-terminal FLAG-tag added for purification purpose, and analyzed by SDS-PAGE (Figures 1B,C). Pure Fg40 protein was initially obtained for the two C1s variants, although at low yields. Indeed, only about 0.2 and 0.3 μg of purified protein per mL of supernatant (SN) were obtained for several purifications of C1sC294R and C1sV316del variants, respectively. This is to be compared with far much higher purification yields (10 μg/ mL SN) for C1sWT. The secreted Fg40 is partially activated (cleaved at the activation cleavage site), leading to a mixture of proenzyme and activated forms: these two forms are resolved in reducing conditions as Fg40 and B bands (Figure 1C), whereas they migrate as a unique Fg40 band in non reducing conditions (Figure 1B), because of the inter-chain disulfide bridge (Figure 1A). Limited variability was observed during multiple purification assays but in all trials Fg40 remained by far the only major band observed.

### Identical Fg40 Fragments Are Obtained for the Two Different pEDS C1s Variants

The Fg40 fragments were purified from the SN expressing the two different pEDS variants and then characterized by N-terminal sequencing and MS analyses. Unexpectedly, these analyses clearly demonstrated that the two samples are identical. Indeed, in both cases, two N-terminal sequences were identified (Figure 2A): the extremity of the B chain (serine protease domain) that is obtained upon C1s activation (IIGGSD…), plus a new N-terminal sequence, observed only in the two Fg40 fragments (CQPVD…). An identical MW of 40.05 kDa was determined by MS for the two variant fragments. This experimental MW corresponds to the 37.9 kDa protein MW calculated from the truncated amino acid sequence, plus a carbohydrate contribution of 2.1 kDa, consistent with the presence of a known glycosylation site at Asn406 (Figure 2A). At this stage, these analyses show that the Fg40 fragments purified from the two different variants correspond unexpectedly to identical C-terminal C1s fragments. Recombinant Fg40 was also directly expressed in HEK293-F cells (Figure 1), with yields of about 0.25 μg/mL of SN after the FLAG-tag affinity purification step.

Intracellularly, the C1s variant proteins are mainly produced as full-length proteins, as it is also the case for C1sWT, but with a slightly reduced production yield (Figure S2). Thus, the Fg40 purified from the SN is very likely a proteolytic fragment, specifically obtained in case of pEDS C1s mutations. Moreover, no strong accumulation of the pEDS C1s variants was observed inside the cells, as could be expected for unfolded proteins.

The structural integrity of CCP1 in C1sWT is maintained by two disulphide bridges (C294-C341 and C321-C354, Figure 2). For both pEDS variants, Fg40 is released by cleavage between K353 and C354. In the native folded CCP1 structure (Figure 2B), this cleavage site is likely kept inaccessible by the C321-C354 disulfide bridge. It can become accessible only when CCP1 locally unfolds, as it is likely the case for the two patient variants, according to the deleterious prediction for these mutations (PROVEAN score, Table 1). The new N-terminus C354 of Fg40 (Figures 2A,C) has lost its C321 counterpart, released after the proteolytic cleavage.

### Comparative Functional Properties of C1s WT and pEDS Variants

Since the unexpected Fg40 protein contains the catalytic serine protease (SP) domain, we have further explored its catalytic properties, as compared to C1sWT. This was performed using purified activated proteins, produced either from the two C1s variants (C1sC294R and C1sV316del) or Fg40 recombinant cell lines, which will be referred to as pEDS-related C1s variants.

We first checked that activation by C1r is unaffected in C1s variants and Fg40, as expected. The pEDS–related variants get indeed fully activated by active C1r, as confirmed by the strong B band in reducing conditions (Figure 1D). An interesting fact is that the purified variants and Fg40 are already partly activated, their level of activation been reproducibly strongly increased as compared to the C1sWT sample (Figure 1C). Such an *in vitro* auto-activation of C1s fragments would be consistent with the previous observation that C1s CCP2-SP fragment became fully activated during crystallization experiments at 20°C, although initially purified in the proenzyme state (16).

**Figure 3:**
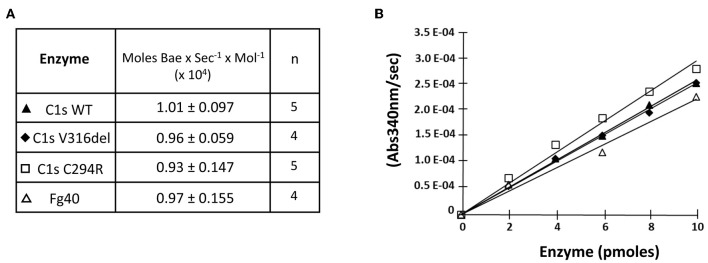
Esterolytic activity of the pEDS C1s variants and Fg40. C1sWT, the two pEDS mutants C1sV316del and C1sC294R, and Fg40 were compared for their ability to hydrolyse N_α_-Benzoyl-L-arginine ethyl ester hydrochloride (BAEe) as described in “Material and Methods.” **(A)** Esterolytic activities are expressed as the number of moles of Bae hydrolysed per second and per mol of enzyme. The mean value and mean deviation of the activities are listed in the table, as well as the number of tests performed (n). **(B)** An intermediate graphical representation of one set of experiments shows more directly that similar slopes of the variation in absorbance at 340 nm vs. enzyme amounts are obtained for the different C1s WT, mutants and Fg40 enzymes.

We then checked if the catalytic activity of all pEDS-related C1s variants was maintained by first measuring the esterolytic cleavage of BAEe. The variants could hydrolyse the substrate with the same efficiency as C1sWT and Fg40, as assessed by the specific activity around 1 x 10^4^ moles of substrate hydrolysed *per sec* and per mole of enzyme (Figure 3). These results strongly highlight the fact that the pEDS variants lead to the secretion of a fragment that retains its native esterolytic activity.

**Figure 4:**
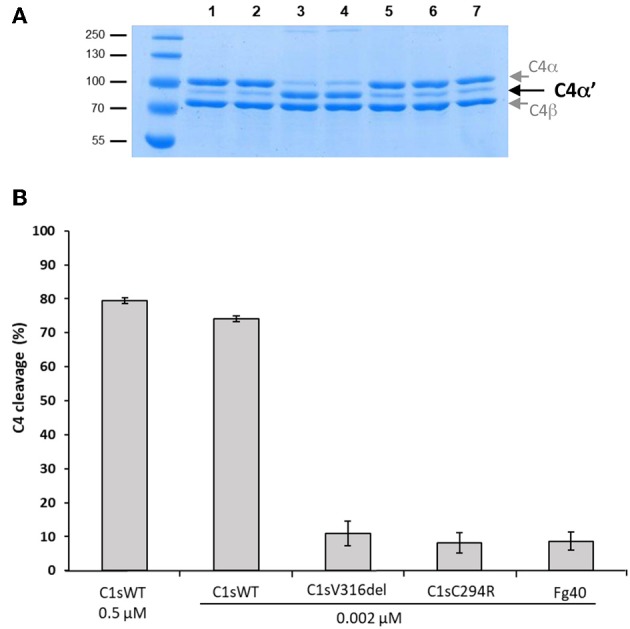
C4 cleavage efficiencies of pEDS C1s variants and Fg40. The C4 cleavage by C1sWT, the two pEDS mutants C1sV316del and C1sC294R and the Fg40 fragment was assessed by incubation of C4 (2.5 μM) with the enzymes at 0.002 μM concentration for 10 min at 37°C. **(A)** SDS-PAGE analysis of C4 (5 μg per well). Lane: 1, non incubated C4; 2, C4 incubated 10 min at 37°C, 3, C4 incubated with 0.5 μM of C1s WT. All the following samples of C4 were incubated with 0.002 μM of enzyme. 4, 5, 6, and 7, with C1sWT, C1sV316del, C1sC294R and Fg40 respectively. C4α' is released by C1s cleavage, C4α and C4β fragments are also labeled. Molecular masses (in kDa) of standard proteins are shown on the left side of the gel. **(B)** C4 cleavage average expressed in % from 3 independent experiments (± mean deviation).

In contrast, the C4-cleavage catalytic activity was dramatically reduced in pEDS-related C1s variants. This is illustrated by the far lower amount of the cleaved C4α' chain that is obtained for the two variants and the Fg40 fragment, as compared to C1sWT (Figure 4, SDS-PAGE lanes 4–7). The C4 cleavage decreased from 75 to 10%. Since CCP1 is missing in Fg40, such a dramatic decrease is fully consistent with our previous observation that both CCP1 and CCP2 are required for an efficient C4 cleavage by C1s (15).

**Figure 5:**
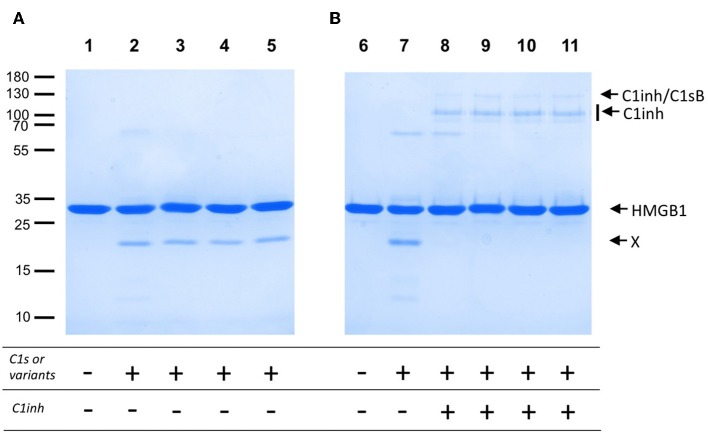
HMGB1 cleavage by pEDS C1s variants and Fg40. HMGB1 (2 μg) was submitted to proteolysis by C1s, WT and pEDS-related variants, with a molar E/S ratio of 4% in 50 mM Tris, 150 mM NaCl, pH 7.4, for 100 min at 37°C. The products were then analyzed by SDS-PAGE in reducing conditions. **(A)** Lanes: 1, HMGB1 with no enzyme, and 2–5, HMGB1 with C1sWT, C1sV316del, C1sC294R and Fg40. X: main primary HMGB1 proteolytic cleavage fragment. **(B)** C1inhibitor (6 pmoles) was preincubated with the enzymes (3 pmoles) for 90 min at 37°C (lanes 8–11). The mixture was then used for proteolytic cleavage of HMGB1 in the conditions of A). 8–11: C1sWT, C1sV316del, C1sC294R and Fg40. HMGB1 alone (6) and HMGB1 with C1sWT (7) are controls. Molecular masses (in kDa) of standard proteins are indicated on the left side of the gel **(A)**.

The lack of efficient C4 cleavage by the two C1s variants and Fg40 opens the question of other possible C1s target(s), with potential links to pEDS disease and symptoms. As a support to this idea, we investigated C1s-mediated HMGB1 cleavage. HMGB1 is indeed a nuclear alarmin exposed during apoptosis, recently revealed to be susceptible to C1s proteolysis (17). We observed a dose-dependent HMGB1 cleavage for C1sWT (not shown), and no apparent differences associated to pEDS variants when we compare the level of the release of the primary HMGB1 cleavage product (Figure 5A). Moreover, this C1s-mediated cleavage of HMGB1 is efficiently inhibited by C1-inhibitor (Figure 5B), which both confirms that it is mediated by C1s and that the C1-inhibitor control is not affected by the two pEDS mutations.

## Conclusion and perspectives

As established here by HEK293 cell transfection experiments, the two patient mutations, namely p.Val316del and p.Cys294Arg, lead to the secretion of a main C-terminal Fg40 fragment of 40 kDa instead of the expected full-length C1s. This fragment results from trypsin-like or C1s-like enzymatic cleavage at the end of the CCP1 module of both variants. Such an unexpected cleavage likely occurs because the two pEDS mutations locally destabilize the surrounding CCP1 module structure, which exposes a new cleavage site after the lysine K353. The observation that two different pEDS mutations lead to an identical molecular outcome reinforces the hypothesis of a likely pathogenic effect for these variants. Ideally, Fg40 fragments would have to be detected in patient material if available in the future. Since Fg40 contains the SP domain, we have checked that its catalytic activity is maintained (Figures 3, 5A) and remains under the control of C1-inhibitor (Figure 5B). Because the N-terminal domain mediating C1s interactions with C1r and C1q is lacking in Fg40, our hypothesis is that the catalytic activity of Fg40 escapes from the physiological control of C1s activity normally applying in the context of the C1 complex.

The dominant effect of these mutations also suggests that such a “free” enzymatic activity, out of C1 complex, might yield a broader spectrum of C1s substrate specificities that will occur in the patient heterozygous context (2). It is indeed important to note that, on the other hand, homozygous deficiencies of early CP components are the strongest genetic risk factors for developing the autoimmune Systemic Lupus, but not periodontitis (18). Therefore, loss of control of complement activation is one possible component of the disease mechanism, as suggested by a parallel study performed on pEDS variants affecting *C1R* (19). Studies on patient fibroblasts would be very useful to definitively validate or invalidate this hypothesis, but we do not have access yet to these very scarce samples. It would be indeed interesting to look at a potential constitutive C4 cleavage in pEDS patient fibroblasts SN, for patients affected by *C1S* mutation, by adding exogenous C4 and looking at its cleavage rate, as shown for some pEDS patients affected by a *C1R* mutation (19). Since the C1s pEDS variants cannot efficiently cleave C4, C4 cleavage would require an intermediate step of Fg40-mediated activation of full-length C1s, produced by the *C1S*WT allele.

Figure 4 clearly shows how the C4-cleavage property of C1s variants and Fg40 is dramatically impaired, which is in line with our previous observations that C1s requires both CCP1 and CCP2 in addition to the SP domain to efficiently cleave C4 (15). This is because the extended C4 recognition domain includes these two CCP modules in C1s, as more recently shown by the X-ray structure of the complex between the homologous MASP2 protease and complement C4 (20). Because the canonical C4 substrate is not efficiently activated by the pEDS C1s variants, we decided to extend the analysis to other non-canonical C1s substrates, starting with HMGB1. We can show that HMGB1 cleavage is not affected for the two patient variants and preserved in the C-terminal Fg40 fragment (Figure 5). Interestingly, C1s-mediated HMGB1 cleavage was noticed to be more efficient in the context of the C1 complex as compared to C1s alone (17), which might thus suggest the production of different HMGB1 fragments, associated to different functional outcomes, although this needs to be further investigated.

HMGB1 is indeed a very potent multifunctional alarmin, and several functions of HMGB1 are known to be modulated by the oxidation state of its cysteines but also by proteolytic cleavage (21, 22). Interestingly, several functional aspects of HMGB1 might be relevant to the periodontal and scarring phenotypes observed in pEDS patients. For example, several studies in rodents and humans suggest a significant role of HMGB1 in periodontal inflammatory infiltration and alveolar bone destruction or resorption (23, 24). Normal human oral mucosa is continuously prone to micro-tissue injury. Several recent studies showed the importance of HMGB1 in normal wound healing *in vitro* and *in vivo*, including both the skin and oral contexts (25–27). HMGB1 is shown to strongly influence sterile scar formation (28). HMGB1 promotes fibroblast migration and mediates fibroblast activity via RAGE (receptor for advanced glycation endproducts)-mediated signaling in keloid scar formation (29). This abnormal fibroblast activity results in excessive synthesis of extracellular matrix compounds, especially collagen. These HMGB1 properties would be relevant to the pEDS heterogeneous symptoms related to abnormal scarring or pretibial plaques. Even recent evidence of brain MRI abnormalities in pEDS patients (30) might be further linked to neuroinflammation properties of HMGB1, although these are ill-defined and need more investigations. More generally, HMGB1 is now considered to orchestrate tissue repair and responses to tissue damage and modulate bone matrix deposition and osseointegration outcomes (31, 32). Thus, HMGB1, and the study of its functional alterations following C1s cleavage, look as relevant targets to further decipher the molecular mechanisms involved in the pEDS syndromes. Investigating other C1s targets, which would be more directly related to proteins classically altered in other EDS syndromes, such as collagens or extracellular matrix proteins, might also provide complementary insights.

## Data Availability Statement

All datasets generated for this study are included in the article/Supplementary Material.

## Author Contributions

VR, IB, and CG designed the study. FD and IB performed the research. IB, VR, CG, and NT analyzed the data. JZ, IK-S, and HS initiated the collaboration on pEDS and, together with RG, AA, and CG, discussed the data and managed the coordination of the French-Austrian studies. AC contributed key reagents. CG wrote the manuscript draft. VR designed the figures. All authors revised and approved the final version of the manuscript.

### Conflict of Interest

The authors declare that the research was conducted in the absence of any commercial or financial relationships that could be construed as a potential conflict of interest.

## Abbreviations

pEDS: periodontal Ehlers-Danlos syndrome
Fg40: C1s C-terminal fragment of 40 kDa uncovered in this study
MW: molecular weight
MS: mass spectrometry
MALDI-TOF: Matrix-Assisted Laser Desorption/Ionization Time-Of-Flight
MRI: Magnetic Resonance Imaging
E/S: Enzyme/Substrate
HMGB1: High mobility group protein B1
SP: conserved serine trypsin-like domain
HMG box: high mobility group box, a conserved protein domain involved in DNA binding
CUB module: as initially found in complement C1r/C1s, Uegf, Bmp1
EGF: calcium binding evolutionary conserved EGF-like domain
CCP: Complement Control Protein evolutionary conserved module (also known as Sushi)
RAGE: receptor for advanced glycation endproducts.
