# Supplementary material for: Two Different Missense C1S Mutations, Associated to Periodontal Ehlers-Danlos Syndrome, Lead to Identical Molecular Outcomes
Source: Front Immunol. 2019 Dec 18;10:2962. doi: 10.3389/fimmu.2019.02962 (PMC6930149; doi:10.3389/fimmu.2019.02962)
Supplement: Supplementary file 1 [file Data_Sheet_1.PDF]

Fig. S1

Primers used for pEDS mutants and Fg40 engineering

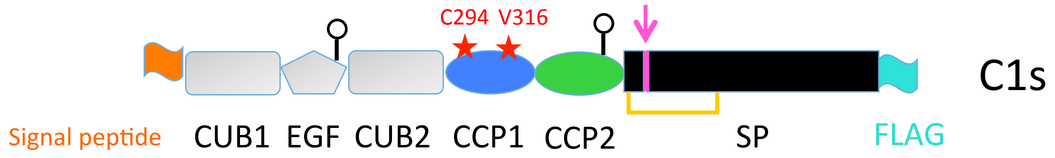

Cloning of pcDNA3.1 C1s WT FLAG

cta gctagc ATG TGG TGC ATT GTC CTG TTT TCA C — *Forward primer*
NheI M W C I V L F S

CCC CGT GAG GAC GAT TAC AAG GAT GAC GAC GAT AAG TAA gaattc aaaggcc — *Reverse primer*
P R E D D Y K D D D D K *EcoRI*

Mutagenesis of pcDNA3.1 C1s C294R

CGC TAT CAT GGA GAT CCA ATG CCG **CGG** CCT AAG GAA GAC ACT CCC — *Forward primer*
R Y H G D P M P **R** P K E D T P

Mutagenesis of pcDNA3.1 C1s V316del

Forward primer

del V

GCA AAA TAT GTC TTT AGA GAT GTG ¦ CAG ATA ACC TGT CTA GAT GGG TTT GAA GTT GTG G
A K Y V F R D V Q I T C L D G F E V V

Cloning of pcDNA3.1 Fg40 by deletion using site-directed mutagenesis

CAC TTT TGG CAT GGG TTT ATG CTT GTC AAC CTG TGG ACT GTG GC — *Forward primer*
L L A W V Y A C Q P V D C G
