# Supplementary material for: Two Different Missense C1S Mutations, Associated to Periodontal Ehlers-Danlos Syndrome, Lead to Identical Molecular Outcomes
Source: Front Immunol. 2019 Dec 18;10:2962. doi: 10.3389/fimmu.2019.02962 (PMC6930149; doi:10.3389/fimmu.2019.02962)
Supplement: Supplementary file 2 [file Data_Sheet_2.PDF]

Fig. S2

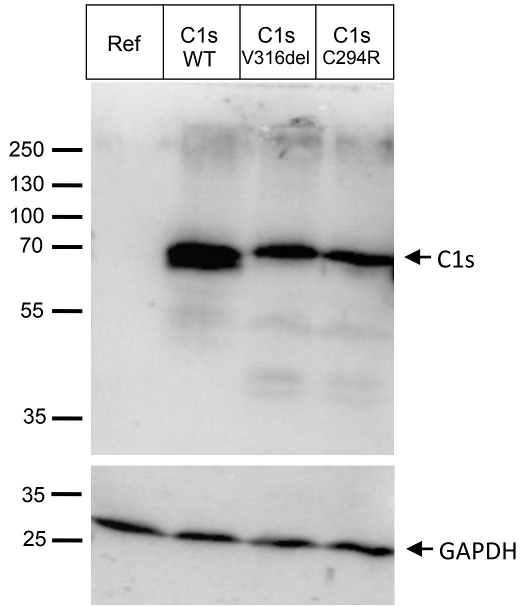

Figure S2. **Cell lysates of HEK293F cells transfected with plasmids coding for C1s WT, V316del and C294R.** Cell were lysed using RIPA buffer (#R0278, Sigma) following the manufacturer protocol. The cell content corresponding to 5 x 10^5 cells was analysed by SDS-PAGE (12.5% acrylamide) under non reducing conditions and then blotted on a nitrocellulose membrane. All samples were analysed with a home-made polyclonal C1s anti-serum (1/ 4,000 dilution) or with an anti-GAPDH (1/5,000; # G99545, Sigma), and further revealed by chemoluminescence using anti-rabbit-HRP (#A0545, Sigma). GAPDH analysis was performed in a second step on the stripped nitrocellulose membrane (Restore TM plus WB Stripping buffer, #46430, Thermofisher, manufacturer protocol). Ref: non-transfected HEK293F cells.
